# A mouse chromosome 4 balancer ENU-mutagenesis screen isolates eleven lethal lines

**DOI:** 10.1186/1471-2156-10-12

**Published:** 2009-03-06

**Authors:** Melissa K Boles, Bonney M Wilkinson, Andrea Maxwell, Lihua Lai, Alea A Mills, Ichiko Nishijima, Andrew P Salinger, Ivan Moskowitz, Karen K Hirschi, Bin Liu, Allan Bradley, Monica J Justice

**Affiliations:** 1 Department of Molecular and Human Genetics, Baylor College of Medicine, Houston, TX 77030, USA; 2 Interdepartmental Program in Cell and Molecular Biology, Baylor College of Medicine, Houston, TX 77030, USA; 3 Cold Spring Harbor Laboratory, Cold Spring Harbor, NY 11724, USA; 4 Center for Molecular and Human Genetics, Columbus Children's Research Institute, The Ohio State University, Columbus, OH 43205, USA; 5 Departments of Pediatrics and Pathology, Institute for Molecular Pediatric Sciences, The University of Chicago, Chicago, IL 60637, USA; 6 Departments of Pediatrics and of Molecular and Cellular Biology, Center for Cell and Gene Therapy, Children's Nutrition Research Center, Baylor College of Medicine, Houston, TX 77030, USA; 7 Wellcome Trust Sanger Institute, Hinxton, UK

## Abstract

**Background:**

ENU-mutagenesis is a powerful technique to identify genes regulating mammalian development. To functionally annotate the distal region of mouse chromosome 4, we performed an ENU-mutagenesis screen using a balancer chromosome targeted to this region of the genome.

**Results:**

We isolated 11 lethal lines that map to the region of chromosome 4 between *D4Mit117 *and *D4Mit281*. These lines form 10 complementation groups. The majority of lines die during embryonic development between E5.5 and E12.5 and display defects in gastrulation, cardiac development, and craniofacial development. One line displayed postnatal lethality and neurological defects, including ataxia and seizures.

**Conclusion:**

These eleven mutants allow us to query gene function within the distal region of mouse chromosome 4 and demonstrate that new mouse models of mammalian developmental defects can easily and quickly be generated and mapped with the use of ENU-mutagenesis in combination with balancer chromosomes. The low number of mutations isolated in this screen compared with other balancer chromosome screens indicates that the functions of genes in different regions of the genome vary widely.

## Background

N-ethyl-N-nitrosourea (ENU) mutagenesis is a powerful tool in the mouse and can be used to generate point mutations in spermatogonial cells. Many ENU screens have been conducted to isolate developmental mutations in a small region of the genome or to isolate mutants with particular defects, reviewed in [1]. In order to examine gene function in targeted regions of the genome, we have taken an approach to mutagenesis screening that does not bias the results towards a particular developmental stage or defect. We have previously conducted forward genetic screens in combination with genetically engineered balancer chromosomes tagged with coat color markers for visual genotyping [2,3]. We previously reported 78 recessive novel lethal mutants that die before three weeks of age (59 were mapped to chromosome 11 and 19 were mapped to chromosome 4) [4,5]. These chromosomes were targeted because of their high conservation with human chromosomes 17 and 1, respectively. In this study, we examine a second region of the distal portion of mouse chromosome 4, located between 96 and 130 Mb. This region is highly conserved with human chromosome 1. By targeting a region with high human conservation, we sought to functionally annotate the genes in the region, and create mouse models of human diseases that map to the corresponding region of human chromosome 1.

## Results

### Chromosome 4 balancer screen

**Figure 1:**
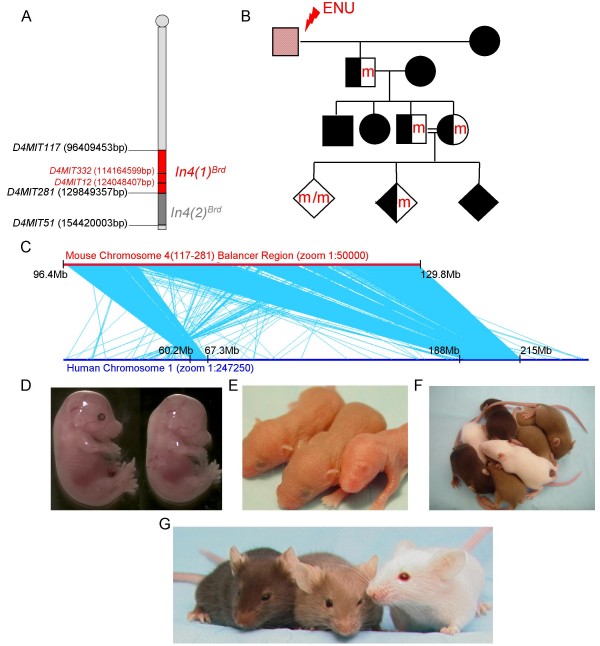
**Chromosome 4 (117–281) Balancer Screen**. **A**. The balancer regions used in this screen (red) and in a previously published screen (dark gray) are shown. Genotyping microsatellite markers are shown in red. Base pairs refer to Ensembl v51. **B**. The mating scheme used for the chromosome 4 (117–281) balancer screen is shown. Red or "m" indicates a new ENU-induced lesion and black indicates the balancer chromosome. Males are depicted with squares and females are depicted with circles. Diamonds represent either sex. **C**. The mouse chromosome 4 balancer is depicted with a red line. Microsyntenies, representing 50–800 bp fragments and averaging 100–200 bp, were blasted and aligned to full-length human chromosome 1, depicted with a blue line. Light blue lines connect each hit of microsynteny conservation between the two chromosomes. Two large areas of linkage conservation are evident on the human chromosome. **D-G**. Homozygous mutants, heterozygous balancer animals, and homozygous balancer animals are all easily distinguished by coat color and the presence or absence of eye pigment at several different stages. A homozygous lethal embryo (no eye pigment) is pictured at E15.5 (**D, right**) next to a control littermate (**D**, **left**). An albino mutant pup lacking eye pigment is easily distinguished from control littermates at P3 (**E, far right**). Homozygous mutants (white, no eye pigment), heterozygous balancer animals (light brown), and homozygous balancer animals (dark brown) are shown at both the P11 (**F**) and adult stages (**G**) and can easily be differentiated by coat color.

We report here the use of a mouse chromosome 4 balancer in an ENU-mutagenesis screen. In this screen, we use an inversion between the simple sequence length repeat markers *D4Mit117 *and *D4Mit281 *that spans a 33 Mb interval containing 470 genes (Ensembl v51) (Fig 1A, C). This screen will be referred to as the chromosome 4 (117–281) screen, named for its flanking microsatellite markers. The balancer interval is highly conserved with 2 distinct regions of human chromosome 1 (60.2Mb-67.3Mb and 188Mb-215Mb) (Fig. 1C). The total genomic length of these two homologous regions, 33Mb, equals that of the balancer region.

Unlike classic *Drosophila *balancer chromosomes, the endpoints of the chromosome 4 (117–281) balancer region do not disrupt any essential genes [6]. Therefore, homozygosity of the balancer is not lethal. The endpoints of the balancer are tagged with the coat color markers *Tyrosinase *(*Tyr*) and *K14-Agouti *(*K14-Ag*). Two copies of the balancer give a dark brown coat color and one copy gives a light brown coat color (Fig. 1F, G) [2]. In the three generation breeding scheme used to isolate recessive mutants, male C57BL/6Brd *Tyr*-/- (B6-albino) are injected with ENU (Fig 1B). B6-albino mice are easily distinguished by coat color from animals carrying the balancer. After injection, fertile B6-albino ENU males are mated to females that are homozygous for the balancer, which is carried on a C57BL/6J *Tyr*-/- genetic background. The G1 progeny from this cross are mated again to homozygous balancer animals to generate G2 animals. Intercrosses of the G2 animals generate homozygous mutant animals in the third generation of the screen. Mice homozygous for the potentially mutated chromosome 4 do not carry the balancer and are therefore albino with a white coat color and a lack of eye pigment (Fig. 1D–G). The lack of eye pigment in the homozygous mutants allowed for visual genotyping of the embryos starting at mid-gestation (E12.5 and beyond) (Fig. 1D). Embryos that were dissected before E12.5 were genotyped by the polymorphic simple sequence length repeat markers *D4Mit12 *and *D4Mit332*, which lie within the balancer region. After birth, coat color (white, light brown, and dark brown) was used to genotype the homozygous mutants, heterozygous balancer mice, and homozygous balancer mice, respectively (Figure 1F, G). Our ability to genotype throughout development and after birth by eye pigment, coat color, or PCR provided a great advantage over traditional mutagenesis screens in determining if phenotypes segregated to the balancer region.

Five hundred and thirty pedigrees were screened for mutations over the chromosome 4 (117–281) balancer. Seventy-five mutant lines were initially identified by the observation of two or more offspring presenting with the same mutant phenotype. The mutant lines displayed a wide range of phenotypes, including lethality, failure to thrive, neurological defects, skeletal defects, craniofacial abnormalities, blood cell defects, urogenital defects, and abnormalities of the skin, coat, and eyes. Only eleven lines segregated in *trans *to the 4 (117–281) balancer and all were lethal. The other 64 mutations are located in chromosome regions outside of the 33Mb balancer region of chromosome 4. Each of these mutant lines are available to the scientific community, and can by found by their MGI Accession number shown in Table 1.

**Table 1:** Mice from lethal lines produced in the chromosome 4 screen

**Line Name**	**MGI Accession #**	**# homozygous balancer (dark brown)**	**# heterozygous mutants (light brown)**	**# homozygous mutants (white)**	**Chi-square probability (p)**
*l4Jus19*	MGI:3033989	29	57	0	p < 0.001
*l4Jus22*	MGI:3038760	40	88	0	p < 0.001
*l4Jus23*	MGI:3043664	18	56	0	p < 0.001
*l4Jus24*	MGI:3043665	39	102	0	p < 0.001
*l4Jus25*	MGI:3046735	46	56	2	p < 0.001
*l4Jus26*	MGI:3577468	38	83	0	p < 0.001
*l4Jus27*	MGI:3577469	34	71	0	p < 0.001
*l4Jus28*	MGI:3577470	30	65	0	p < 0.001
*l4Jus29*	MGI:3577471	24	40	2	p < 0.001
*l4Jus30*	MGI:3577472	15	27	4	p < 0.04
*l4Jus31*	MGI:3577474	40	82	17	p < 0.002

The genotypes of progeny resulting from intercrosses between animals heterozygous for both the lethal mutation and the balancer are shown. Genotypes were determined at weaning by coat color, and a chi-square analysis was performed for each line to determine if the observed genotype ratio was statistically different from the expected Mendelian ratio.

### Eleven lethal lines isolated

Eleven lethal lines named *l4Jus19*, and *l4Jus22 to l4Jus31 *segregated in *trans *to the 4 (117–281) balancer, and therefore, map to the region (Table 1). A lethal mutant line was detected and mapped simultaneously to the region if 30 homozygous or heterozygous balancer mice were observed at weaning with no homozygous mutant littermates. Additional matings confirmed the heritable nature of the mutation as well as the segregation of the mutation to the balancer region (Table 1). The eleven lines fell into ten complementation groups. The two lines *l4Jus19 *and *l4Jus26 *fail to complement and are likely different mutant alleles of the same gene (Table 2). In the cross between *l4Jus19 *and *l4Jus26*, 31 mice carrying one or two copies of the balancer were found at weaning while no animals lacking the balancer, and therefore having an albino coat color, were observed (Expected = 8, p < 0.001).

**Table 2:** Complementation Analysis of Chromosome 4 (117–281) Lethal Lines

Line name	*l4Jus19*	*L4Jus22*	*l4Jus23*	*l4Jus24*	*l4Jus25*	*l4Jus26*	*l4Jus27*	*l4Jus28*	*l4Jus29*	*l4Jus30*
*l4Jus22*	+									

*l4Jus23*	+	+								

*l4Jus24*	+	+	+							

*l4Jus25*	+	+	+	+						

*l4Jus26*	**-**	+	+	+	+					

*l4Jus27*	+	+	+	+	+	+				

*l4Jus28*	+	+	+	+	+	+	+			

*l4Jus29*	+	+	+	+	+	+	+	+		

*l4Jus30*	+	+	+	+	+	+	+	+	+	

*l4Jus31*	+	+	+	+	+	+	+	+	+	+

The results of complementation testing of the lethal mutants are shown. Crosses that complement are indicated by a '+' sign, and crosses that fail to complement are indicated with a '-' sign. The only lines that fail to complement are *l4Jus19 *and *l4Jus26*. For this cross 31 mice were examined at weaning, and no albino animals were observed (p < 0.001).

The genotypes of all animals that were alive at weaning are shown for the eleven lines in Table 1. For all of the lines, non-Mendelian ratios of the expected genotypes, due to embryonic or postnatal lethality, were observed. For seven of the lines (*l4Jus19*, *l4Jus22*, *l4Jus23*, *l4Jus24*, *l4Jus26*, *l4Jus27*, and *l4Jus28*), no homozygous mutants were ever observed at birth or weaning, indicating fully-penetrant embryonic lethality. For three lines (*l4Jus25*, *l4Jus29*, and *l4Jus30*), a few homozygous mutants were observed at weaning. It is possible that these weanlings are survivors of incompletely penetrant phenotypes or that the mutated gene is outside of but linked to the balancer region, and that these weanlings are recombinant wild-type mice. However, we would expect recombinants to be healthy and of normal size, and these mice were unhealthy and died soon after weaning, making the first possibility more likely.

Notably, seventeen small *l4Jus31 *homozygous mutants were present out of 139 mice weaned but none survived to sexual maturity, indicating that this line had a postnatal time of death (Table 1, 3).

**Table 3:** Determining the Time of Death of Chromosome 4 (117–281) Lethal Mutants

**Line Name**	**# Litters examined**	**Stages examined**	**# Homozygous mutants**^a^	**# Heterozygous mutants or balancer monozygotes**	**# Resorbed embryos**	**Unable to determine**^b^	**Stage of death**	**Notes on mutants**
*l4Jus19*	1	E12.5	2	2	8	0	E9.5–12.5	Dead at E12.5
*l4Jus22*	1	E11.5	0	7	1	0	Not determined	
*l4Jus23*	4	E11.5–12.5	0	21	16	0	Before E11.5	
*l4Jus24*	6	E7.5–13.5	0	23	11	1	Before E7.5	
*l4Jus25*	3	E10.5, E12.5	3	17	7	0	E9.5–12.5	Smaller, cardiac defects
*l4Jus26*	6	E8.5–12.5	5	30	15	1	E5.5–7.5	Smaller, developmental delay
*l4Jus27*	8	E7.5–12.5	0	38	5	4	Before E8.5	
*l4Jus28*	15	E8.5–10.5, E12.5	25	89	3	0	E9.5–12.5	Vascular defects
*l4Jus29*	2	E8.5, E12.5	3	11	2	0	Before E7.5	Fail to grow after implantation
*l4Jus30*	7	E10.5–12.5	16	45	5	0	E9.5–12.5	Craniofacial defects
*l4Jus31*	8	E9.5, E12.5, P11–25	15	36	6	0	Post-natal	Neurological defects

^a ^After E12.5 mutant embryos were identified by lack of eye pigment and heterozygous mutants or balancer homozygotes were identified by eye pigmentation

^b ^Embryos were lost during the dissection

**Figure 2:**
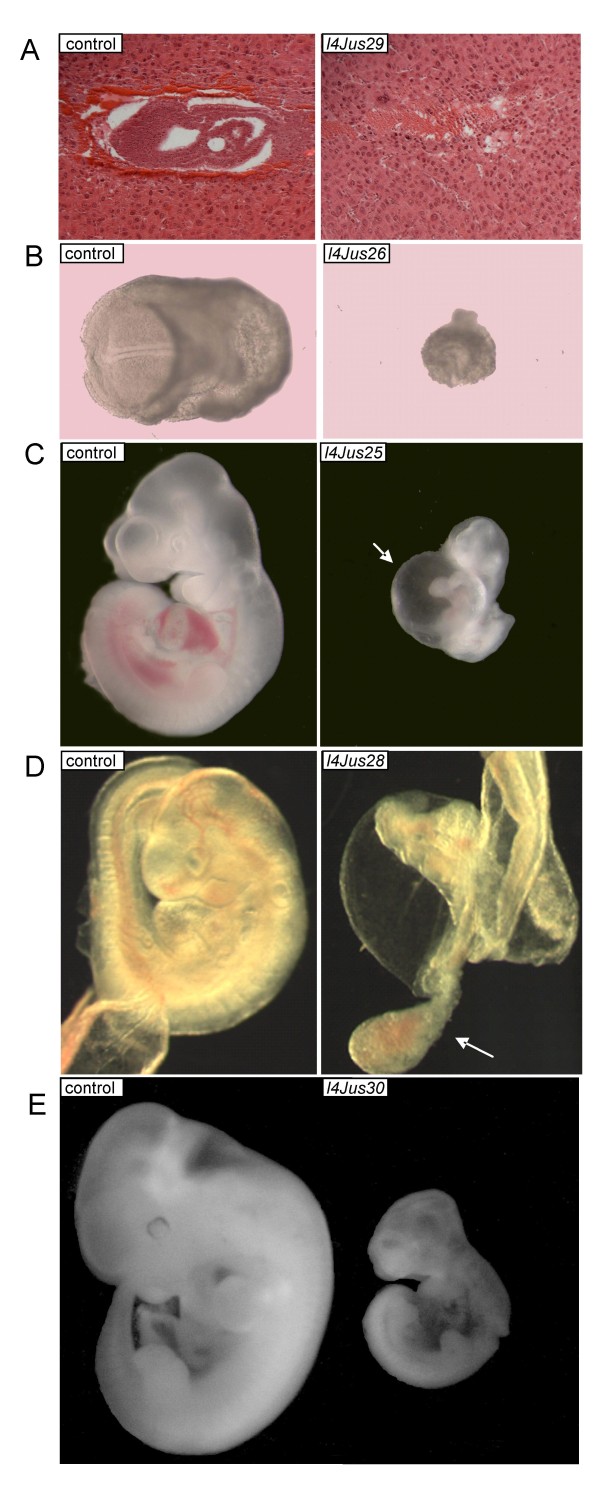
**Chromosome 4 (117–281) Lethal Mutants**. Mutant embryos are pictured on the right and littermate controls are pictured on the left. All of the images of mutants and control from the same line were taken at the same magnification except D in which the mutant was photographed at a higher magnification in order to better visualize the allantois. **A**. Hematoxylin and Eosin stained sections of a presumed *l4Jus29 *homozygous mutant (right) and control littermate (left) are pictured at E7.5. The mutants die prior to gastrulation. Because of lack of embryonic tissue, these mutants were not genotyped but were identified by abnormal histology. **B**. *l4Jus26 *at E8.5. Mutants are smaller than control littermates and exhibit developmental delay. **C**. *l4Jus25 *at E10.5. Mutants display cardiovascular defects and are smaller than control littermates. Pericardial edema is evident in the mutant and is indicated with a white arrow. **D**. *l4Jus28 *at E9.5. Mutant embryos have not turned and the allantois grows but does not fuse with the chorion, shown with a white arrow. **E**. *l4Jus30 *at E11.5. Mutant embryos are smaller than control littermates and exhibit growth defects, as well as abnormal telencephalon development.

We carried out timed matings and embryo dissections in order to determine the time of death of ten mutant lines (Table 3). We were unable to determine the time of death of one mutant line. We observed five mutant lines with homozygotes that die between embryonic days (E) 5.5 and 8.5 (*l4Jus23*, *l4Jus24*, *l4Jus26*, *l4Jus27*, and *l4Jus29*). For *l4Jus23*, *l4Jus24 *and *l4Jus27*, no homozygous mutants were observed, although resorbed embryos were noted, suggesting a time of death earlier than the stages examined (E11.5–E12.5, E7.5–E13.5, and E7.5–E12.5, respectively). Homozygous mutants from *l4Jus26 *were smaller than littermate controls and arrested prior to E7.5 (Fig. 2B). Presumed homozygous mutants from *l4Jus29 *are dying at E7.5, but were unable to be genotyped due to lack of embryonic tissue. Histological sections of these presumed *l4Jus29 *embryos at E6.5 revealed that the embryos induce the formation of a maternal decidua but fail to grow after implantation, and must die prior to gastrulation (Fig. 2A).

We observed four mutants that exhibited lethality during the E9.5–E12.5 developmental time period (*l4Jus19*, *l4Jus25*, *l4Jus28*, and *l4Jus30*). *L4Jus19 *homozygous mutants are smaller, dead, and necrotic by E12.5. Homozygous mutants from *l4Jus25 *displayed arrested development and cardiovascular defects, including pericardial edema, at E10.5 (Fig. 2C). Homozygous mutants from *l4Jus28 *are lethal at E10.5. This was evident in some of the mutants that were smaller or had not turned at this time point, and had head, cardiac and vascular defects. Additional embryo dissections at E9.5, revealed very abnormal allantoids, and their failure to fuse with the chorion to establish the maternal-fetal circulation, which is likely to result in embryonic death (Fig. 2D). Mutants homozygous for *l4Jus30* were often smaller than their littermates and displayed abnormal growth and telencephalon development at E11.5 (Fig. 2E).

**Figure 3:**
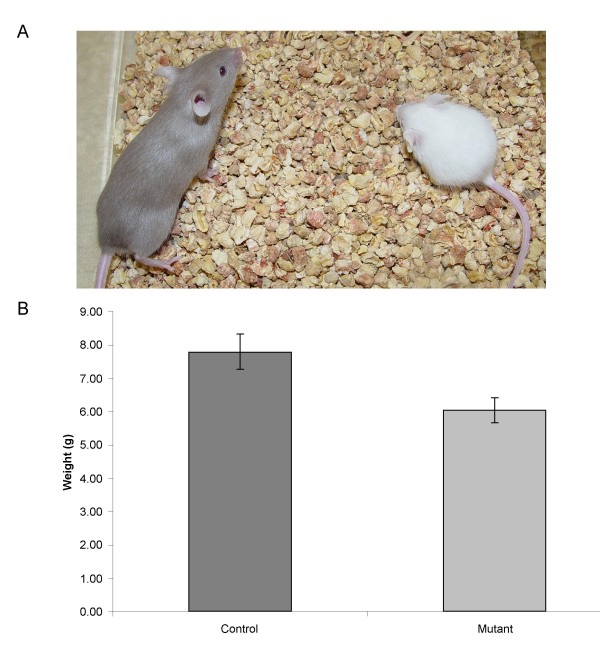
***l4Jus31 *mutants are smaller than control littermates**. **A**. The homozygous mutant is confirmed by the white coat color and is pictured on the right. The heterozygous littermate, with a light brown coat color, is pictured on the left. The picture was taken at postnatal day 28. **B**. *l4Jus31 *homozygous mutants are significantly smaller than their control littermates at P14 (Student's t-test, n = 5 mutants, 4 control animals, p < 0.001).

Only one of the eleven lethal mutant lines, *l4Jus31*, exhibited postnatal lethality. Several homozygous mutants were observed at 2 – 4 weeks after birth. Mutants did not appear to differ in size from wild-type littermates at or before birth. However, mutants are significantly smaller than their littermates (Student's t-test p < 0.001, Fig. 3) by P14 and exhibited neurological defects, including hyperactivity, ataxia and seizure (Additional file 1). *L4Jus31 *homozygous mutants never survived to sexual maturity. We conducted complete blood counts (CBCs) to determine if homozygous mutants displayed any blood defects but found no significant differences compared to control littermates (data not shown).

It is likely that the majority of the mutations in our eleven lethal lines do not lie in genes that have been previously knocked out, because only 30 of the 470 genes in the region have published knockouts with lethality before three weeks of age [7-42]. However, by comparing the phenotypes, two candidate genes emerged (Table 4). In both the knockout of *T-cell acute lymphocytic leukemia 1 *(*Tal1*) and *l4Jus25*, mutant embryos are smaller than expected and display pericardial edema [9], making *Tal1 *a candidate for *l4Jus25*. One interesting candidate gene for *l4Jus31 *is *Disabled homolog 1 *(*Dab1*). The *l4Jus31 *mutant is very similar to the knockout mouse in that both display postnatal lethality after a few weeks of birth and have neurological defects, including ataxia [10,11]. We designed primers that would amplify all of the coding exons as well as the exon-intron boundaries of both genes and sequenced *Tal1 *in *l4Jus25*, and *Dab1 *in *l4Jus31 *(Additional File 2). However, we were unable to detect any mutations in the coding regions of either gene. This suggests that these lines contain lesions in other genes in the region, or represent new alleles of genes previously knocked out. It does not rule out the possibility that these mutations lie in regulatory regions for these genes.

**Table 4:** Previously published knockout alleles in the chromosome 4 (117–281) balancer

**Gene**	**Published phenotype**
**Pre-implantation**	

*Cdca8* *Cdc20* *Faf1*	embryos present at E3.5 but fail to form fully expanded blastocyst [8] embryos arrested at the two cell stage [39] embryos arrested at the two cell stage [12]
*Nasp*	at E3.5, blastocyts are smaller with fewer cells [13]

**E5.5–E8.5**	

*Foxd3*	failure to gastrulate, finger-like extensions seen at distal tip [14]
*Gjb3*	lethality before somite formation [15,16]
*Hdac1*	embryonic lethality before turning of embryo, growth retarded after E7.5 [17,18]
*Macf1*	lethality before somite formation [19]
*Ncdn*	resorption was evident prior to E6.5 [20]
*Urod*	lethality before somite formation, no fetuses at E7.5–E9.5 [21]

**E9.5–E12.5**	

*Bmp8b*	smaller than expected, delayed, die by E9.25 [22]
*Ppap2b*	abnormal gastrulation, dead by E10.5 [23,24]
*Slc2a1*	prenatal lethality E10–E14, reduced embryo size, overall developmental delay [25]
*Stil*	lethality around E10.5, abnormal left-right axis patterning, growth retardation abnormal neural tube morphology and development [26]
*Tal1*	lethality at E8.5–E10.5, arrested development, cardiovascular defects, defects in
	hematopoiesis, distended pericardial sacs, pale and necrotic [9]

**E13.5–E18.5**	

*Dhcr24*	some die before birth, others smaller with less adipose tissue, decreased cholesterol levels, male and female infertility [27]
*Gjb5*	death at E11.5–E14.5, smaller with placental defects [28]
*Marcksl1*	neural tube [29]
*Mtf1*	embryos die around E14 due to liver degeneration [30]
*Ssbp3*	prenatal lethality, abnormal head development [31]
*Tie1*	death before E13.5, respiratory, cardiovascular, skin defects [32]
*Ybx1*	lethality E13.5-just after birth, reduced embryo size, craniofacial defects, abnormal neural tube closure [33]

**Peri-natal**	

*Jak1*	neonates smaller with abnormal B and T cell development [34]
*Khdrbs1*	2/3 die at birth, and the survivors live well into adulthood [35]
*Nfia*	lethality at P0, survivors have severe brain abnormalities [36]
*Pou3f1*	die within a few hours of birth due to respiratory defects [37]
*Ror1*	die just after birth with abnormal blood chemistry and respiratory system defects [38]
*Slc6a9*	die just after birth with severe motor and respiratory defects[40]

**Post-natal**	

*Dab1*	lethality at 20–30 days, decreased leptin levels, nervous system defects including ataxia, abnormal brain morphology [10,11]
*Dmbx1*	lethality before P9, smaller, little or no milk in stomachs [7,41,42]

## Discussion

The eleven novel lethal lines isolated in the 4 (117–281) screen represent a diverse group of phenotypes, which can be used as a resource for the biomedical community to investigate developmental processes. Our results suggest that the majority of the mutations isolated are likely in genes that have not been previously targeted by mutation, or are novel alleles of previously characterized genes. Only two mutants (*l4Jus25 *and *l4Jus31*) showed phenotypes similar to known knockouts, but we could not detect a molecular lesion in either candidate gene. The chromosome 4 mutations may be valuable models for human birth defects or neurological disease.

Here we have implicated additional loci in development which may otherwise go undiscovered, because ENU mutagenesis using a balancer is without bias towards a particular abnormality or time of death. Targeting a particular region of the genome using a balancer chromosome as described here provides an advantage over genome-wide mutagenesis followed by rapid mapping using single nucleotide polymorphism (SNP) arrays. Using a balancer, lethal mutations can be identified quickly by the lack of albino animals seen at weaning, the lethal lines are immediately mapped upon isolation to the balancer region, and then they can be easily maintained because they are not lost by recombination and can be followed by coat color.

By screening only 530 families or pedigrees for mutation, the 4 (117–281) screen is not meant to approach saturation, but instead, provides a sampling of gene function in a region. In the mouse, each three generation pedigree takes approximately nine months to breed, and 530 pedigrees represents nearly 16,000 mice. Thus, the numbers of families that can be examined for mutation are limited by cost and time. However, we expected to isolate more than 11 mutant lines in this limited screen, because of our extensive experience in using balancers productively for ENU screens. Previously, we carried out a screen of another portion of chromosome 4 distal to the 4 (117–281) region described in the current report, which is located between *D4Mit281 *to *D4Mit51 *(referred to as 4 (281–51); Fig 1A). In the 4 (281–51) screen, a total of 23 mutant lines were isolated, even though a similar number of pedigrees were examined (551; Table 5). Nineteen lines were homozygous lethal, two lines had growth defects (*gro69 *and *gro81*), one had neurological defects (*nur61*), and one had eye defects (*eye02*) [4]. The difference in the numbers of mutations isolated in these two screens is statistically significant (Table 5, p < 0.05), using a Chi Square test for homogeneity of samples. This test compares numbers drawn from a binomial distribution (mutant or not mutant), which are sampled from more than two populations and may fall into multiple classes (mutant phenotypes). We also compared the numbers of mutants isolated from these two screens on mouse chromosome 4, against a third screen carried out on mouse chromosome 11. In the mutagenesis screen using *In(Trp53-Wnt3)8Brd *on chromosome 11, 59 lethal mutations and 32 mutations that produced other phenotypes for a total of 91 mutations were isolated in 785 pedigrees screened [5]. The chromosome 11 *Trp53-Wnt3 *interval is predicted to contain 905 genes, chromosome 4 *D4Mit117–D4Mit281 *is predicted to contain 470 genes and the *D4Mit281–D4Mit51 *interval 461 (Table 5). The homogeneity of samples test shows that we isolated significantly more mutants in the chromosome 11 screen than in either of the two chromosome 4 screens. This difference cannot be accounted for by gene density alone. It is possible that more gene families are present on chromosome 4 than on chromosome 11, making genes in the 4 (117–281) region functionally redundant. However, a Pfam database survey reveals that about 50% of the genes in each of the three regions belong to gene families. Another possibility is that many of the ENU induced mutations on chromosome 4 were not detrimental or had very subtle phenotypes missed in the original screening process. Our phenotype survey was focused on developmental defects, so it was not comprehensive. The poor mutation recovery might indicate that the *D4Mit117-D4Mit281 *region contains genes that require a challenge or stress, such as bacterial or viral infection, for their function to be revealed. There is precedence for such a requirement on chromosome 4. Just proximal to *D4Mit117*, the lipopolysaccharide (*LPS*) gene causes no phenotype when deleted unless the mice are challenged with a bacterial infection, in which case they die [43]. Finally, genetic background may play a role in the observation of phenotypes. The chromosome 11 screen was carried out on a mixed background of C57BL/6J and 129S6/SvEvTac. However, the screens on chromosome 4 were carried out on a predominantly C57BL/Brd *Tyr*-/- background, making the possibility of phenotype suppression in a mixed background less likely. A control for the mutagenesis treatment and for the phenotype screening methods is the cohort of mutations that mapped to other chromosomes. Of note, a similar number of mutations that segregated genome-wide were isolated in each of these screens (Table 5; ). These mutations had a wide variety of phenotypes, but none were lethal, because we did not wish to recover lethal mutations segregating genome-wide.

**Table 5:** Comparison of Chromosome 4 and 11 ENU Balancer Screens

**Parameter**	**Chr 4** **(*117–281*)**	**Chr 4** **(*281–51*)**	**Chr 11** **(*Trp53-Wnt3*)**
Interval Breakpoints	96.4–129.8 Mb	129.8–154.4 Mb	69.4–103.7 Mb

Interval size	33 Mb	25 Mb	34 Mb

Number of annotated genes	470	461	905

Pedigrees screened	530	551	785

Lethal lines	11	19	59

Other viable phenotypes	0	4	32

Total mutations	11	23	91

Mutations/Mb	0.33	0.92	2.68

Homogeneity of samples^a^	3.913, p < 0.05	22.8, p < 0.001	37.2, p < 0.001

Frequency of mutations mapping outside of the balancer region	1 in 5.4	1 in 4.8	1 in 4.2

Literature Reference	This report	Hentges *et al. *2006 [4]	Kile *et al. *2003 [5]

In all cases, the phenotype screen included a coat color screen for all lethals in the balancer region. It also included a complete blood count for all blood cell parameters, a screen for neutral cholesterols using tandem mass spectrometry, a urine screen for albumin and glucose, and a general visual assessment of skin, coat, eyes, fur, movement and balance, as well as morphology on all viable mice, which would carry mutations segregating to the balancer region or elsewhere in the genome.

^a ^A Chi square test for homogeneity of samples in a binomial distribution with one degree of freedom. The values represent the significance from the numbers of mutations isolated in the next column, with the exception that the number in column c is the difference from column a.

## Conclusion

We have already shown that mouse chromosome 11 is unique because it contains a high number of essential genes, and we have suggested that genes with similar functions may be clustered in the genome [44]. Such an idea is not novel, since essential genes are clustered in other organisms, including *C. elegan*s and *Drosophila *[45]. After screening 530 families for the chromosome 4 (117–281) region, it became obvious that ours was not a productive screen for functionally annotating this region with developmental phenotypes. We would therefore not recommend screening additional families for mutations in the 4 (117–281) region for lethality or for the developmental phenotypes listed in materials and methods. Instead, alternative phenotype assays may uncover the functions of genes in this region. Ultimately, once the genes responsible for the mutant phenotypes are found, we will have a greater understanding of gene function on mouse chromosome 4 and will have the tools necessary for understanding human diseases that map to human chromosome 1.

## Methods

### Mutagenesis Screen

The balancer chromosome B6-Brd.*In(D4Mit117;D4Mit281)1Brd *or *In4(1)*^*Brd *^was used to isolate recessive mutations in a three generation screen after ENU treatment of B6-albino males. The coat color scheme for carrying out mutagenesis and the balancer mice have been previously described [2,4]. Five hundred and thirty pedigrees were screened and eleven lethal lines were isolated. All experiments involving animals had ethical approval from the Institutional Animal Care and Use Committee (IACUC) of Baylor College of Medicine. The balancer chromosome was created and maintained on the on the C57BL/6-*Tyr*^*c*-*Brd *^albino strain background by intercrosses.

The phenotype screen included a coat color screen for all lethals in the balancer region. It also included a complete blood count for all blood cell parameters using a Cell-Dyn 3500R (Abbott Laboratory), a screen for neutral cholesterols using tandem mass spectrometry, a urine screen for albumin and glucose (Chemstrip9 from Roche), and a general visual assessment of skin, coat, eyes, fur, movement and balance, as well as morphology on all viable mice, which would carry mutations segregating to the balancer region or elsewhere in the genome.

### Sequence comparisons

Genomic sequences of mouse and human were downloaded from Ensembl v.49 . Each region of mouse sequences was divided into 150-kb fragments, which were then blasted using Megablast . The sequence comparison was carried out on a Sun cluster with SunFire 280R . Mouse genomic annotation was downloaded from Ensembl BioMart v.49 . To visualize the blast results, we developed in-house software written in Microsoft Active Server Page . All blast results were uploaded in a MS SQL server database, and the results displayed on a PC.

### Complementation Analysis

Mice that were heterozygous for both the lethal mutations and chromosome *In4(1)*^*Brd *^balancer were bred together. If albino mice were observed at weaning, the two lines complemented. If no albino mice in a cohort of more than 30 were found at weaning, the lines failed to complement.

### Embryo dissection and analysis

For each mutant line, timed matings were carried out on heterozygous animals. Mating cages consisting of one male and two females were set up in the evenings and females were observed for a vaginal plug for five consecutive mornings. The day that a vaginal plug was observed was considered to be E0.5.

Initial embryo dissections were performed on E12.5 and then earlier or later, depending on the findings. All embryos that were obtained from dissections were genotyped, and many were photographed with a Leica microscope from Diagnostic Instruments or a Zeiss microscope. Genotyping on embryos before E12.5 was performed by PCR using DNA prepared by incubating yolk sacs or whole embryos with 50 mM NaOH at 95°C for 20 minutes, followed by neutralization with 1/5 volume of 0.5 M Tris, pH 8.0. The PCR conditions were: 1× Invitrogen PCR buffer, 1.5 mM MgCl_2_, 0.3 mM dNTPs, 0.5 μM *D4Mit12 *(GCTTGCTTTAGGAGTGTGCC and TATTTGCTCTCCATTTCCCC) or *D4Mit332 *(TCAATCCCATTGGCTATATATGC and TGAGAAACCTCTCCAGCACC) primer mix, 250 ng template, 0.25 U Taq Polymerase (Invitrogen, Carlsbad, CA). Cycling conditions were: 94°C 5 min; 40 cycles of 94°C 45 sec; 58°C 45 sec; 72°C 45 sec; then 7 min at 72°C; followed by incubation at 4°C. After E12.5, embryos could be genotyped visually by the presence or absence of eye pigment.

### Histology

Decidua or embryos were dissected at E6.5–E12.5, fixed for 3 hours in Bouin's fixative or overnight in 4% PFA, and then dehydrated with series of ethanol washes and embedded in paraffin. The embryos were sectioned at 5–7 μm, staining with Hematoxylin and Eosin, and analyzed with a Zeiss Axioplan light microscope.

### Comparison of ENU-mutants with previously published knockouts

Mouse Genome Informatics was used to find candidate genes for the 11 novel ENU-mutants on chromosome 4 between 96–130 Mb. The Phenotypes, Alleles and Disease Models database was searched in this region for published knockouts . Ninety-seven of the 470 genes in the region have been targeted by gene trap or knockout constructs. In searching for knockouts that were lethal, we excluded genes that were lethal after three weeks of age, or if there was a double or triple knockout required for lethality. Using this method, we found 30 alleles that are homozygous lethal before weaning and thus are potential candidate genes in the region. The phenotypes of these knockout lines and the comparisons of these with our lethal lines have been summarized in Supplemental Table 1.

### Sequencing

Coding exons of *Tal1 *and *Dab1 *were identified using Ensembl v50 and primers were designed to flank each exon by 100–150 bp (Additional file 2). Genomic DNA from *l4Jus25 *and *l4Jus31 *was phenol-chloroform extracted from frozen livers. PCR amplicons were either shipped to Agencourt Bioscience Corporation (Beverly, MA) for single pass sequencing with both forward and reverse PCR primers or were sequenced directly using BigDye Terminator v3.1 (Applied Biosystems) according to the manufacturer's instructions. The sequencing chromatograms were analyzed using Sequencher 4.7 (Ann Arbor, MI).

### Protein Family Analysis

The protein family data are based on Pfam annotated databases. The most current Pfam is version 23.0 [46].

## Authors' contributions

MKB and BMW performed all timed matings, embryo dissections, and genotyping, took most of the pictures and movies, sequenced candidate genes, and prepared the manuscript. AM helped with embryo dissections and genotyping. LL carried out embryo dissections, genotyping and photographing of *l4Jus28*. AAM, IN, and AB created the chromosome 4 balancers and critically reviewed the manuscript. APS supervised the balancer chromosome screen, mutant line maintenance, complementation analysis, and helped with pictures and movies. BL carried out the alignment of the *D4Mit117-D4Mit281 mouse *balancer region to other species. IM helped with sectioning and histology and critically reviewed the manuscript. KKH critically reviewed the manuscript. MJJ conceived of the study, obtained funding for the study, helped to dissect embryos, compared the balancer region mutagenesis data and helped to draft the manuscript. All authors read and approved the final manuscript.

## Supplementary Material

Additional file 1 ***L4Jus31***** has neurological defects**. This movie shows a homozygous *l4Jus31 *mutant (white coat color) that has an abnormal gait with both hyperactivity and ataxia when compared to a control littermate (homozygous balancer mouse with a dark brown coat color). The homozygous *l4Jus31 *mutants are smaller than their littermates. Click here for file

Additional file 2 **Primers used for sequencing of Tal1 and Dab1.** Coding exons of *Tal1 *and *Dab1 *were identified using Ensembl v50 and primers were designed to flank each exon by 100–150 bp. Click here for file
